# Short-term results of interventional therapy for infants (7–36 months old) with patent ductus arteriosus and moderate-to-severe pulmonary hypertension: a retrospective study

**DOI:** 10.1186/s13019-020-01110-5

**Published:** 2020-04-22

**Authors:** Yue Shu, Yilong Guo, Xiaoqi Wang, Dexing Zhou

**Affiliations:** Department of Cardiovascular Surgery, The Second Affiliated Hospital of Hainan Medical University, 48th of Bai Shui Tang Road, Haikou, Hainan People’s Republic of China 570311

**Keywords:** Patent ductus arteriosus, Pulmonary arterial hypertension, Infants, Interventional therapy, Congenital heart disease

## Abstract

**Background:**

Patent ductus arteriosus (PDA) is a common congenital heart disease. Interventional therapy is an important treatment for PDA. Nevertheless, few studies have investigated the safety and effectiveness of interventional therapy for infants (age, 0–36 months) with PDA and moderate-to-severe pulmonary hypertension. Therefore, this study aimed to analyze the short-term (6 months) results and interventional therapy experience for infants with PDA and moderate-to-severe pulmonary hypertension.

**Methods:**

Clinical records, echocardiographic data, and angiocardiography data of 28 infants (age, 7–36 months) who underwent interventional therapy for PDA and moderate-to-severe pulmonary hypertension between December 2011 and January 2017 at our hospital were retrospectively analyzed. All infants were treated using an Amplatzer occluder with local and deep sedation anesthesia under radiographic guidance.

**Results:**

Infants with PDA and moderate-to-severe pulmonary hypertension had poor growth. Trace residual shunts were found in two infants immediately after procedure; both had disappeared by 6 months after procedure. No significant interventional therapy-related complications occurred in the other cases. Pulmonary systolic pressure, left atrial dimension, and left ventricular end-diastolic dimension immediately after interventional therapy and 6 months later were lower than the preoperative levels (*P* < 0.05). The left atrial and left ventricular end-diastolic dimensions at 6 months after interventional therapy were smaller than those immediately after interventional therapy (*P* < 0.05). Pulmonary systolic pressure rates immediately after interventional therapy and 6 months later were not significantly different (*P* = 0.505). Moreover, there were no significant differences in the left ventricular ejection fraction before, immediately after, and at 6 months after interventional therapy (*P* = 0.628).

**Conclusions:**

For infants (age, 7–36 months) with PDA and moderate-to-severe pulmonary hypertension, interventional therapy can achieve excellent immediate and short-term (6 months) results with careful preoperative evaluations, strict operative procedures, and careful follow-up.

## Background

Patent ductus arteriosus (PDA) is a common congenital heart disease that accounts for 10–21% of congenital heart diseases [1]. Interventional therapy is an important treatment for PDA. Nevertheless, there are few studies of the safety and efficacy of interventional therapy for infants (0–36 months old) with PDA and moderate-to-severe pulmonary hypertension. Therefore, this study was designed to analyze the short-term (6 months) results and interventional therapy experience for infants (7–36 months old) with PDA and moderate-to-severe pulmonary hypertension.

## Methods

### Patients

From December 2011 to January 2017, a total of 78 infants with PDA were successfully treated with interventional therapy at our hospital. The inclusion criteria were as follows: age ≤ 36 months and PDA associated with moderate-to-severe pulmonary hypertension (pulmonary hypertension was determined by the ratio of pulmonary to aortic systolic pressure). Pulmonary and aortic systolic pressure were measured directly using a cardiac catheter before PDA occlusion (mild: 0.25–0.45; moderate: 0.46–0.75; severe: > 0.75) [2]. The exclusion criteria were other cardiovascular malformations that required simultaneous treatment and cardiac insufficiency or Eisenmenger syndrome. Finally, 28 infants were included.

### Preoperative preparation

Electrocardiography (ECG), chest X-ray, and transthoracic echocardiography and blood gas analysis were routinely performed for all infants before interventional therapy.

### Operative methods

**Fig. 1:**
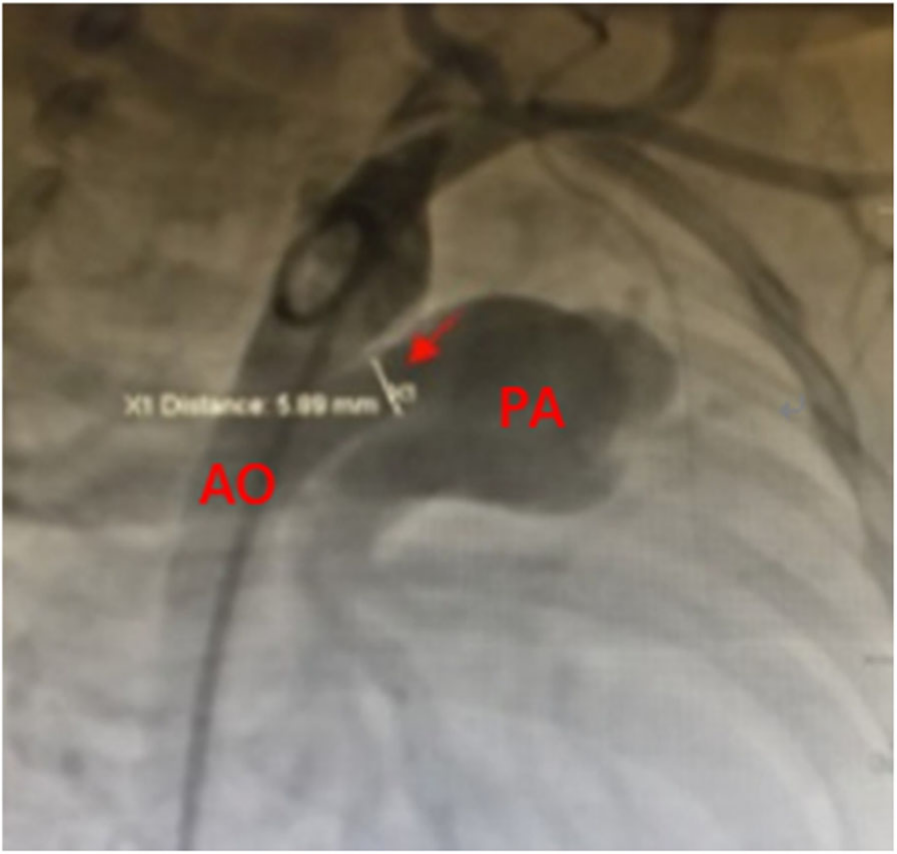
Aortography prior to occlusion. The red arrow indicates the patent ductus arteriosus with a diameter of 5.89 mm. AO, aorta; PA, pulmonary artery

**Fig. 2:**
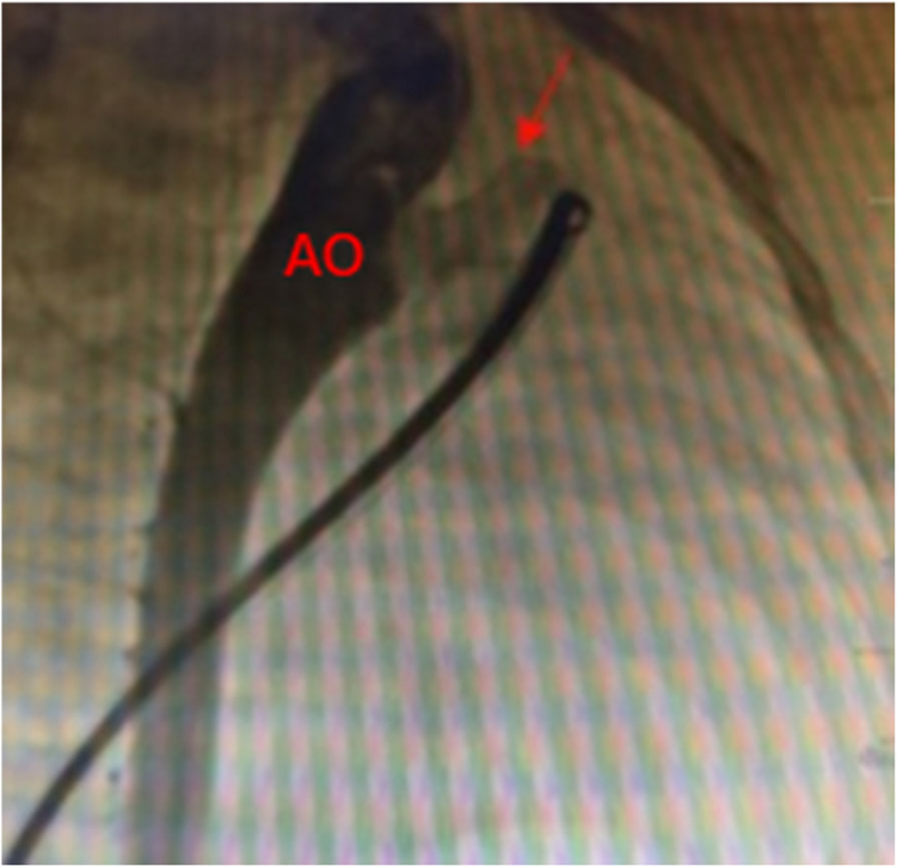
Aortography results after occlusion. The red arrow indicates the occluder. There was no residual shunt after occlusion. AO, aorta

Procedure was performed under local and deep sedation anesthesia. Deep sedation anesthesia is mainly achieved by the intravenous administration of ketamine and midazolam. No muscle relaxants were used, and autonomous respiration was maintained. Catheterization of the right femoral artery and vein was routinely performed. Pressures of the pulmonary artery (PA), ascending aorta, and descending aorta (DA) were measured immediately thereafter. Subsequently, aortography was performed to determine the location, size, and type of the PDA and to determine whether there were any other malformations (Fig. 1). Depending on the angiography results, an appropriate PDA Amplatzer occluder (Starway Medical Technology Inc., Beijing, China) and sheath were selected to occlude the PDA. Pressures of the PA, ascending aorta, and DA were monitored after the occluder was inserted appropriately. After 10 min, aortography was performed again (Fig. 2). The following conditions were confirmed before releasing the occluder: pulmonary systolic pressure decreased after occlusion; pressure gradient < 20 mmHg between the proximal and dismal parts of the aorta at the location of the occluder; only mild or trace residual shunt after interventional therapy (trace: extravasation of the contrast medium has a cloudy appearance at the PA side of the PDA; mild: the PA, but not the pulmonary valve, could be seen using radiography; moderate: both the PA and valve could be seen using radiography) [3]; and vital signs were stable during procedure.

### Follow-up

Transthoracic echocardiography, chest X-ray, and ECG and blood and urine tests were routinely performed at 24 h after procedure. Every infant underwent transthoracic echocardiography performed by a physician in our department at 6 months after procedure.

### Data collection and processing

Preoperative data collected included the sex ratio, age, weight, height, body mass index, PDA diameter, PDA diameter/DA diameter, PA/DA systolic pressure, PDA classification, left atrial (LA) diameter, left ventricular end-diastolic dimension (LVEDD), and left ventricular ejection fraction (LVEF). The pulmonary and aortic systolic pressures during the preoperative and immediately postoperative periods were measured using a cardiac catheter. LA, LVEDD, pulmonary systolic pressure, and LVEF were measured for every infant using transthoracic echocardiography at 6 months after procedure.

### Statistical analysis

Data are expressed as means ± standard deviation (SD) or percentages. Between-group comparisons of perioperative variables were analyzed using the chi-square test, Fisher’s exact test, Student’s t test, or Mann-Whitney U test, as appropriate. All statistical data were analyzed using SPSS version 19.0 (IBM Inc., Armonk, NY, USA). *P* < 0.05 was considered statistically significant.

## Results

### Characteristics of the study population

**Fig. 3:**
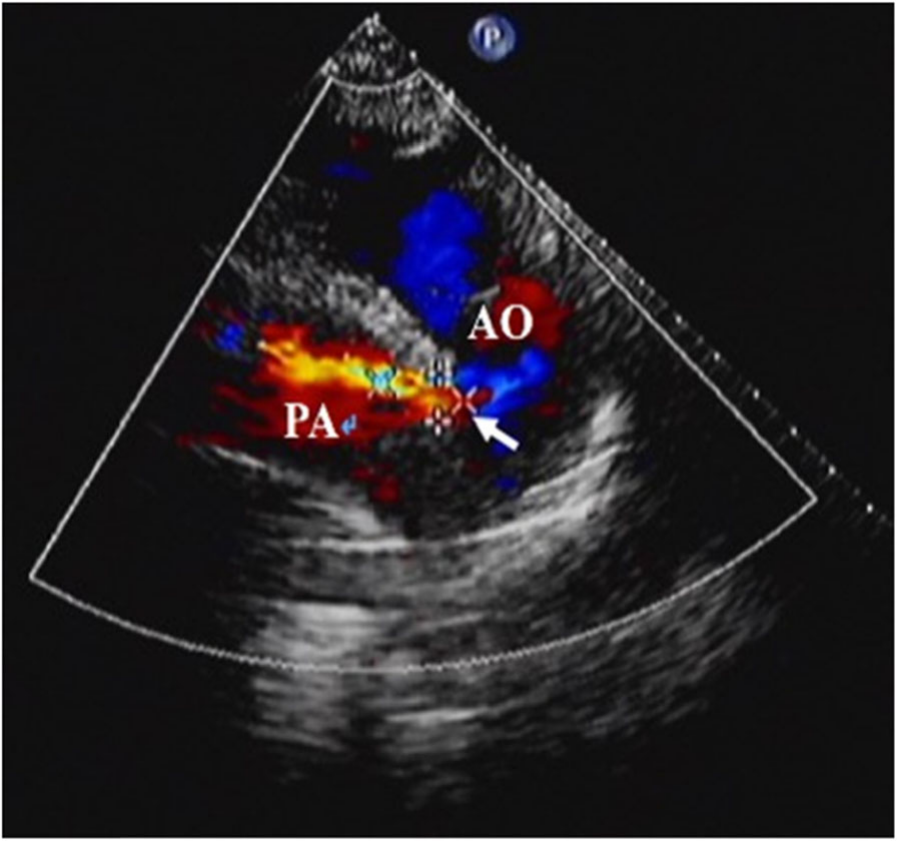
Transthoracic echocardiography results before occlusion. The white arrow indicates the patent ductus arteriosus. PA, pulmonary artery; AO, aorta

There were 28 infants in this study. The height and weight of every infant were collected and compared with those of infants in the normal cohort. According to the World Health Organization child growth and development standard 2006, their weights and heights were lower than those of the normal cohort (*P* < 0.05) [4]. Therefore, their growth was poor. Because the infants were too young and their blood vessels were too narrow, it may not be sufficient to describe the size of the PDA as the diameter of the narrowest portion of the vessel (PDA diameter) alone. Therefore, the size of the PDA was described as the PDA and PDA/DA diameters. The DA diameter refers to the diameter of the DA at the origin of the PDA. Characteristics of the study population are shown in Table 1. Transthoracic echocardiography results before occlusion are shown in Fig. 3.

**Table 1:** Characteristics of the study population

Patients (n)	28
Female	18 (64.29%)
Age (mean ± SD, months)	25.50 ± 10.54
Weight (kg)	9.73 ± 3.04
Height (cm)	84.39 ± 10.26
BMI (kg/m^2^)	13.40 ± 2.53
PDA diameter (mm)	3.86 ± 1.44
PDA diameter/DA diameter (%)	43.30 ± 15.90
PA systolic pressure/DA systolic pressure	0.565 ± 0.023
PDA classification (n)	
Tube type	3
Funnel type	25
LA diameter (mm)	23.57 ± 3.74
LVEDD (mm)	37.21 ± 6.07
LVEF (%)	55.46 ± 1.95

BMI, body mass index; PDA diameter, the diameter of the narrowest part of the PDA; DA, descending aorta; PA, pulmonary artery; LA, left atrium; LVEDD, left ventricular end-diastolic diameter; LVEF, left ventricular ejection fraction.

### Intraoperative data

Compared to the preoperative measurements, the pulmonary systolic pressure immediately after interventional therapy was lower (*P* < 0.01) and the aortic systolic pressure was higher (*P* = 0.042). Preoperative and immediately postoperative pulmonary and aortic systolic pressures are shown in Table 2. Immediately postoperatively, the pressures of the proximal and distal parts of the aorta at the location of the occluder were 119.54 ± 19.31 mmHg and 123.57 ± 18.616 mmHg, respectively, and there was no significant difference between them (*P* = 0.429). There were only two infants who had trace residual shunt immediately after interventional therapy, which disappeared after 6 months. In this study, only six infants developed ecchymosis at the site of femoral artery puncture, which disappeared within 1 week after procedure. No thrombus, pseudoaneurysm, and other vascular damage or complications occurred.

**Table 2:** Pulmonary and aortic systolic pressures (mean ± SD, mmHg) during the preoperative and immediately postoperative periods

	Patients	Pulmonary systolic pressure	Aortic systolic pressure
Preoperative	28	62.89 ± 12.37	110.32 ± 13.25
Immediately postoperative	28	27.82 ± 7.85	119.54 ± 19.31
P	–	< 0.01^b^	0.042^a^

Statistical comparisons: ^a^*p* < 0.05; ^b^*p* < 0.01.

### Short-term results

**Fig. 4:**
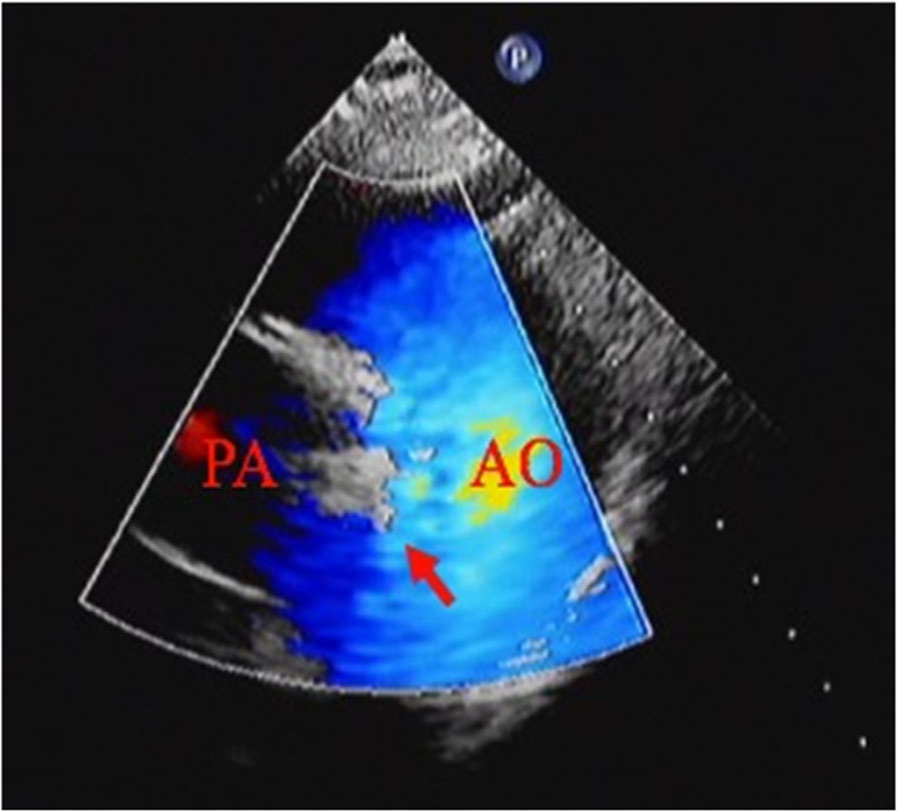
Transthoracic echocardiography results after occlusion. The red arrow indicates the occluder. There was no residual shunt after occlusion. PA, pulmonary artery; AO, aorta

Transthoracic echocardiography was performed for every infant at 6 months after interventional therapy (Fig. 4). Pulmonary systolic pressure, LA diameter, and LVEDD immediately postoperatively were lower than the preoperative dimensions (*P* < 0.05). The LA diameter and LVEDD at 6 months after procedure were smaller than the immediate postoperative dimensions (*P* < 0.05). The results showed that the diameter of the left side of the heart decreased within 6 months after procedure. There were no significant differences in the pulmonary systolic pressures at 6 months after procedure and immediately after procedure (*P* = 0.505). There were also no significant differences in the LVEF before, immediately after, and at 6 months after procedure (*P* = 0.628). There was only one infant whose pulmonary systolic pressure was 65 mmHg immediately after procedure. After 6 months of regular treatment with bosentan (2 mg/kg, twice per day), the pulmonary systolic pressure decreased to 30 mmHg. Characteristics of the infants during the perioperative period are shown in Table 3.

**Table 3:** Characteristics of infants during the perioperative period (mean ± SD)

	Preoperatively	Immediately postoperatively	At 6 months postoperatively	P
LA diameter (mm)	23.57 ± 3.74^b^	20.21 ± 4.63^a^	18.04 ± 2.15 ^a,b^	< 0.01
LVEDD (mm)	37.21 ± 6.07^b^	32.82 ± 5.37^a^	29.82 ± 4.39 ^a,b^	< 0.01
Pulmonary systolic pressure (mmHg)	62.89 ± 12.37^a,b^	27.82 ± 7.85^a^	25.57 ± 5.17^b^	0.013
LVEF (%)	55.46 ± 1.95	55.43 ± 1.71	54.18 ± 9.46	0.628

LA, left atrium; LVEDD, left ventricular end-diastolic dimension; LVEF, left ventricular ejection fraction. Statistical comparisons: ^a^*p* < 0.05; ^b^*p* < 0.01.

## Discussion

The main findings of this study were that the growth of infants in this study was poor because of PDA and moderate-to-severe pulmonary hypertension and that pulmonary systolic pressure decreased after procedure. Only one patient experienced moderate pulmonary hypertension immediately after procedure; that patient recovered after 6 months of regular treatment with bosentan. Furthermore, the LA diameter and LVEDD of all infants decreased by the 6-month follow-up and eventually returned to normal, which was consistent with the results of a previous study [5].

The ductus arteriosus is the connection between the main PA and the distal part of aortic arch; it serves as the main pathway of fetal blood circulation. Commonly, the ductus arteriosus functionally closes within 10–20 h after birth and becomes the arterial ligament during the neonatal period. PDA refers to the persistent opening of the ductus arteriosus after birth, which is one of the most common congenital heart diseases that often occurs in infants born prematurely [6]. Clinical features of infants with PDA are recurrent respiratory tract infections, feeding difficulties, and heart failure; therefore, treatment should be performed as early as possible after diagnosis [7].

Compared with traditional thoracotomy, interventional therapy has three major advantages: less trauma without thoracotomy; interventional therapy can be performed under local and deep sedation anesthesia without tracheal intubation; and quick recovery after procedure. Therefore, interventional therapy has become an important treatment for PDA.

### Treatment experience

#### Preoperative experience

Chest X-ray, transthoracic echocardiography, and ECG and femoral arterial blood gas analysis were routinely performed before interventional therapy [8]. Interventional therapy was performed when the participants met the following criteria: comorbidities that contraindicate interventional therapy, including pneumonia, were excluded by chest X-ray; transthoracic echocardiography showed enlargement of the left atrium and ventricle (indicating that the volume load of the left side of the heart was increased and left-to-right shunt was present), LVEF was > 50%, and other cardiovascular malformations that require simultaneous treatment were excluded; malignant arrhythmias were excluded by ECG; and differential cyanosis was excluded by a femoral arterial blood gas analysis.

#### Intraoperative experience

##### Target vessel catheterization

All patients were infants with low weights, and target vessel catheterization was difficult to perform. To improve the success rate of catheterization, the following suggestions should be considered: all procedures should be performed in a satisfactory state of anesthesia; the fasting duration should be reduced (4 h before procedure); intravenous fluids should be administered before interventional therapy; and catheterization should be performed under ultrasound guidance if necessary.

##### Strict operative procedures

All infants had low weights, fragile vascular states, and cardiac tissues. Therefore, interventional therapy should be performed cautiously to avoid bleeding or malignant arrhythmia caused by any rupture of the blood vessels or heart.

##### Careful selection of the occluder and sheath

Because the femoral artery and vein of infants are small, a large sheath or occluder may not reach the correct location and may cause serious damage to the vessel and endocardium [9, 10]. In our experience, the sheath is directly proportional to the weight of the infant and the diameter of the PDA. The sheath diameter is slightly larger than the weight (kg) of the infant. For infants with moderate-to-severe pulmonary hypertension, the left-to-right shunt is reduced due to the decreased pressure gradient. The PDA is muscular only during infancy, when it is more elastic. Therefore, the diameter of the selected occluder should be larger than measured diameter [11]. In our department, we choose occluders that are 4–6 mm larger than the measured diameter. The largest sheath and occluder used in this study were 10 Fr and 12–14 mm, respectively, for an 18-month-old girl whose weight was 8.7 kg and PDA diameter was 7.1 mm.

##### Trial occlusion

All infants need to undergo trial occlusion. During this trial, the infant’s vital signs need to be monitored. When the vital signs of the infant are not stable, the trial should be stopped and the occluder should be retracted immediately. The nature of the pulmonary hypertension should be determined. For patients with PDA and pulmonary hypertension, the nature of the pulmonary hypertension is determined via right heart catheterization and pulmonary vasoreactivity testing using oxygen inhalation. Sometimes, the diagnosis needs to be confirmed further using a lung biopsy. In this study, enlargement of the left side of the heart and adequate blood oxygen saturation suggested dynamic pulmonary hypertension. Therefore, the evaluation process was simplified and the nature of pulmonary hypertension was determined directly by trial occlusion. When the pulmonary systolic pressure was reduced > 25% after the PDA was occluded, we concluded that the patient had dynamic pulmonary hypertension and interventional therapy was performed [2, 12]. Iatrogenic descending aortic coarctation should be avoided. Pressures of the ascending and descending aortas should be monitored during trial occlusion. Previous studies showed that iatrogenic descending aortic coarctation was not present when the pressure gradient between these two areas was < 20 mmHg after the PDA was occluded [13, 14]. Therefore, when the blood flow is clearly blocked or when the pressure gradient is > 20 mmHg, the occluder should be retracted. The shape and location of the device should be monitored via aortography 10 min after trial occlusion. When the location of the occluder is not stable and its shape is not satisfactory, the occluder should be retracted. The occluder should be retracted when the status of the residual shunt is greater than mild.

#### Postoperative experience

The common complications of interventional therapy for PDA are lower extremity veno-occlusive or ischemic necrosis, hemolysis, thrombocytopenia, residual shunts, and device displacement [15]. To reduce the incidence rates of these complications, certain steps should be performed after procedure. First, the duration of lower limb immobilization and pressure dressing after interventional therapy should be limited. The pulse of the dorsalis pedis artery should be monitored, and lower limb edema should be prevented. Second, urine should be checked for hemolysis at 24 h after interventional therapy; treatment may be performed with hormones and alkalization of the urine when hemolysis is present. In severe cases, the occluder should be removed surgically [16]. Third, unless there are contraindications to antiplatelet therapy, aspirin should be prescribed routinely for 6 months (3–5 mg/kg/d).

##### Follow-up

Transthoracic echocardiography, chest X-ray, and ECG and blood and urine tests should be performed at 24 h after procedure. Transthoracic echocardiography should be repeated for every infant at 6 months after procedure.

##### Limitations

This study had some limitations. This was a retrospective, non-randomized study. No multivariate analyses were performed. Therefore, we could not eliminate selection bias. Further prospective, randomized, large-scale, long-term studies that include multivariate analyses are needed to validate our findings.

## Conclusions

It can be challenging to provide interventional therapy for infants (age, 7–36 months) with PDA and moderate-to-severe pulmonary hypertension. However, with careful preoperative evaluation, strict operative procedures, and careful follow-up, interventional therapy can achieve excellent immediate and short-term (6 months) results in these infants.

## Data Availability

All data generated or analyzed during this study are included in this published article and in the supplementary information files.

## Abbreviations

PDA: Patent ductus arteriosus
ECG: Electrocardiogram
DA: Descending aorta
BMI: Body mass index
LA: Left atrium
LVEDD: Left ventricular end-diastolic dimension
LVEF: Left ventricular ejection fraction
SD: Standard deviation
